# Severe coronary artery spasm during left atrial appendage closure plus catheter ablation for atrial fibrillation: case presentation

**DOI:** 10.1186/s12872-022-02483-2

**Published:** 2022-02-11

**Authors:** Xin Xie, Zijun Chen, Yu Luo, Xiaorong Li, Jian Zhou, Jinbo Yu, Bing Yang

**Affiliations:** Department of Cardiology, Shanghai East Hospital, School of Medicine, Tongji University, Shanghai, 200092 China

**Keywords:** Coronary artery spasm, Atrial fibrillation, Catheter ablation, Left atrial appendage closure, Case report

## Abstract

**Background:**

Left atrial appendage closure (LAAC) combined with radiofrequency catheter ablation (RFCA) as a hybrid procedure is commonly performed to treat atrial fibrillation (AF). Although this treatment carries a low risk of coronary artery spasm (CAS), and has never been observed in LAAC procedure, caution still needed to be taken. We presented a case of CAS that occurred in an AF patient during the hybrid procedure.

**Case presentation:**

The patient was a 65-year-old man with paroxysmal AF who developed CAS during RFCA and LAAC. In this case, LAAC was performed ahead of RFCA. After atrial septal puncture, the occluder was advanced into left atrium through delivery sheath, and successfully deployed in the LAA. After verifying the assessment of “PASS” criteria, we decided to release the device. However, before releasing the occluder in LAAC, the patient’s blood pressure (BP) fell to 70/45 mmHg with heart rate (HR) drop and ST-segment elevation in II, III, and aVF and reciprocal ST-segment depression in I and aVL. Isotonic sodium chloride load was administered. After 3 min, the BP and HR raised, and ST-segment returned to normal. The occluder was successfully released after the stable condition of the patient. Then, RFCA was sequentially performed. When isolating the right pulmonary veins, the patient’s BP and HR fell again with ST-segment elevation in inferior leads. Spontaneous ventricular tachycardia and fibrillation developed rapidly and defibrillation was performed immediately with success. Coronary angiography revealed the obstruction of the right coronary artery which disappeared completely after intracoronary nitroglycerin injection (1 mg). Under systemic diltiazem infusion, the RFCA procedure was accomplished. After the procedure, the patient recovered without any neurologic deficit, and CAS has never recurred with isosorbide mononitrate treatment during follow-up.

**Conclusions:**

CAS is a rare complication associated with AF hybrid procedure. Attention should be paid to this rare but potentially life-threatening complication.

## Background

The combination of left atrial appendage closure (LAAC) and radiofrequency catheter ablation (RFCA) as a hybrid procedure for the treatment of symptomatic atrial fibrillation (AF) is technically feasible [1]. Although the risk of causing coronary artery spasm (CAS) is low, this treatment still needs to be taken with caution [2]. CAS refers to severe reversible diffuse or focal vasoconstriction, which is featured as ischemia with no obstructive coronary artery [3]. CAS has recently been reported as a complication of AF ablation [2]. Severe CAS during AF hybrid procedure may lead to hemodynamic collapse. This rare but potentially life‐threatening complication must be recognized and treated promptly. However, CAS during AF hybrid procedure has never been reported. Described below is a case of angiographically confirmed coronary vasospasm following LAAC plus RFCA for atrial fibrillation.

## Case presentation

**Fig. 1:**
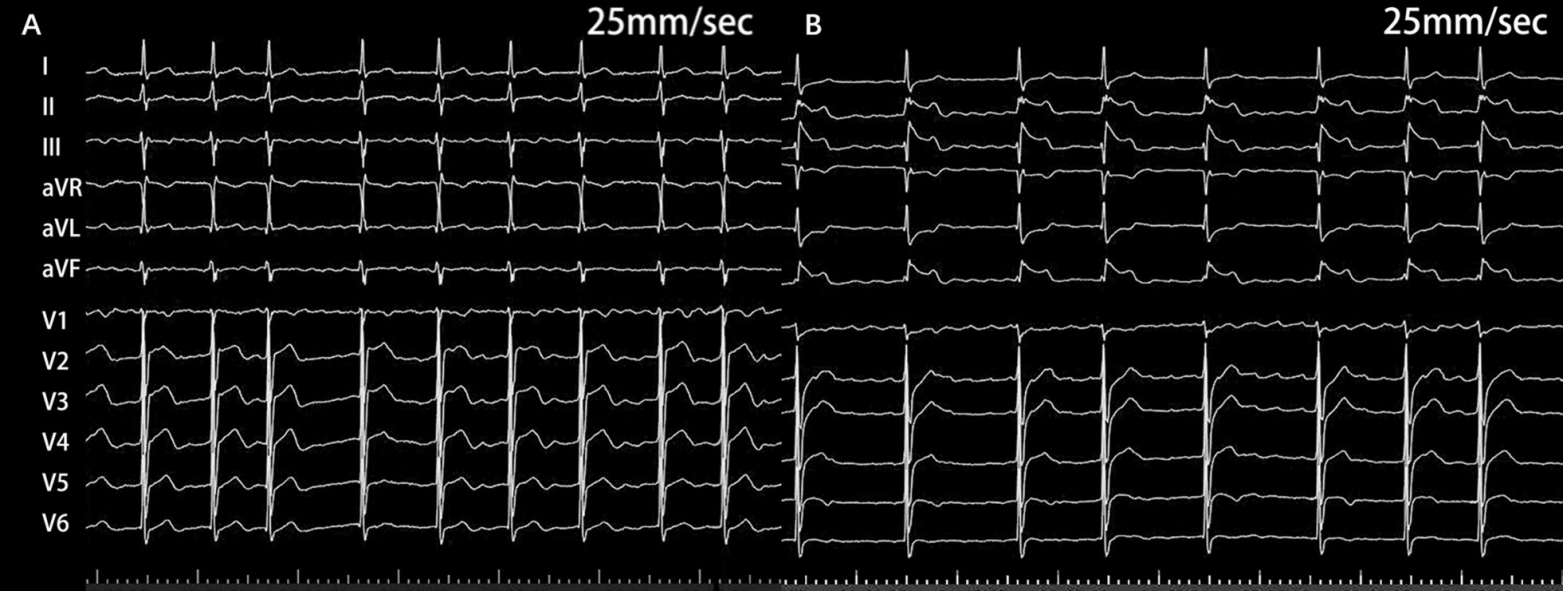
Twelve-lead electrocardiography. **A** Preoperative electrocardiography; **B** electrocardiography with ST-segment elevation in II, III aVF and reciprocal ST-segment depression in I, aVL

**Fig. 2:**
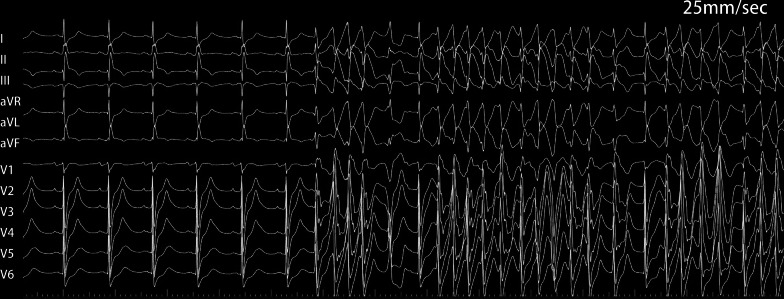
Twelve-lead electrocardiography. Ventricular tachycardia occurred during ablation

**Fig. 3:**
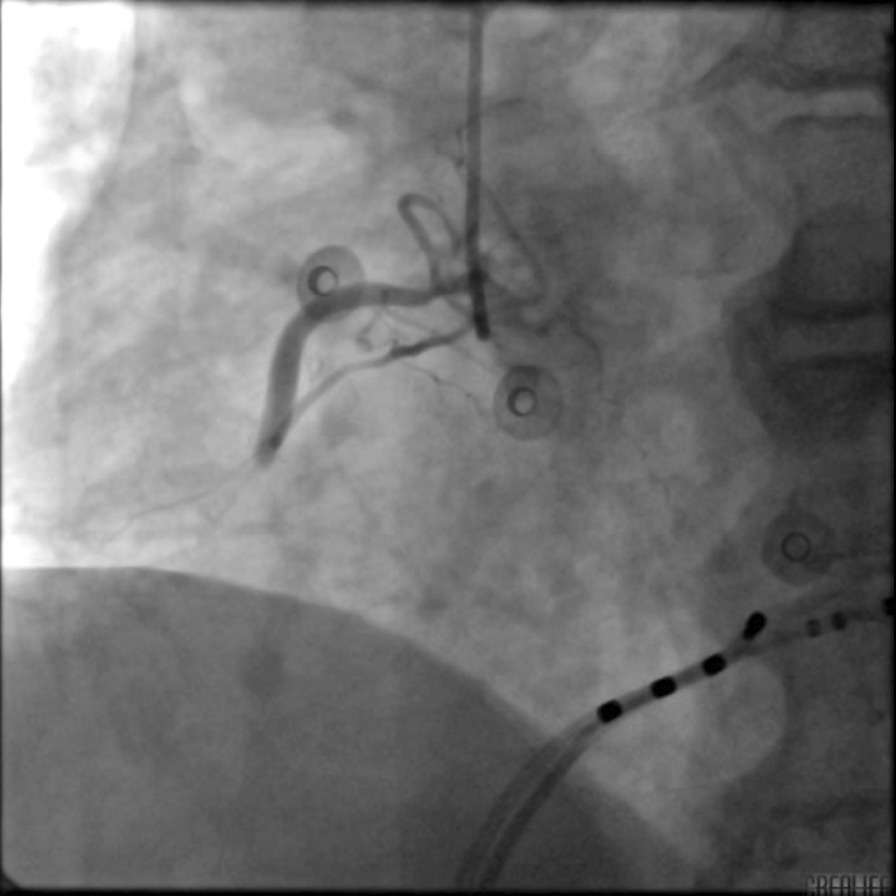
Coronary angiogram. An obstruction in the midportion of right coronary artery

**Fig. 4:**
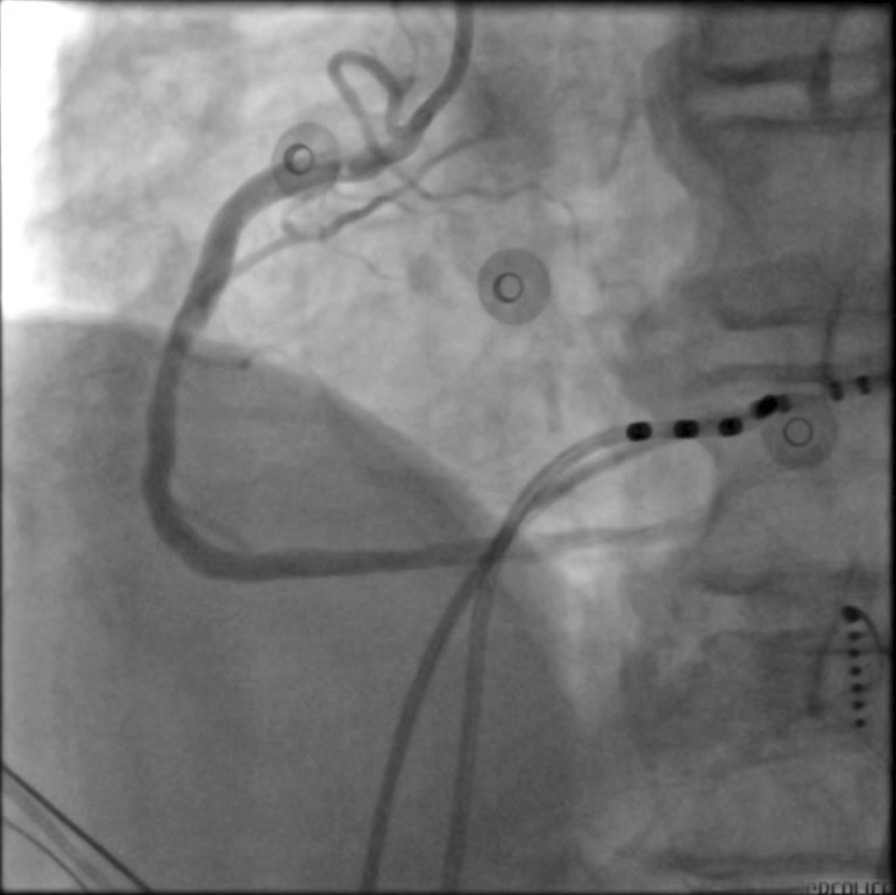
Coronary angiogram. Relief in right coronary artery obstruction after intracoronary injection of nitroglycerin

A 65-year-old man with paroxysmal AF was admitted for LAAC and RFCA. The CHA_2_DS_2_-VASc score was 3 owing to hypertension, peripheral artery disease, and age. Computed tomography angiography revealed no significant coronary artery stenotic lesions. Transoesophageal echocardiography (TEE) demonstrated no intracardiac thrombus. General anesthesia was maintained with propofol and remifentanil. Following transseptal punctures, intra-venous heparin was administered to maintain an activated clotting time of more than 300 s. A suitable Watchman device (Boston Scientific) was first performed with the guidance of TEE. After verifying the assessment of “PASS” criteria, we decided to release the device. However, before releasing the device, the patient’s blood pressure (BP) fell to 70/45 mmHg with heart rate (HR) drop and ST-segment elevation in II, III, and aVF and reciprocal ST-segment depression in I and aVL (Fig. 1). An isotonic sodium chloride load was administered immediately. Cardiac tamponade was ruled out by TEE. After 3 min, the BP and HR raised, and ST segment returned to normal. The patient's condition was stable after an observation for 30 min. Thus, after reconfirming suitable positioning of the device and good plugging effect, the occluder was released and RFCA was performed orderly. When isolating the right pulmonary veins, the patient’s BP and HR dropped again with ST-segment elevation in inferior leads. Spontaneous ventricular tachycardia and fibrillation developed rapidly (Fig. 2) and defibrillation was performed immediately with success. Immediate coronary angiography showed total occlusion of the right coronary artery (Fig. 3), no air bubbles were noted in coronary angiography, and no thrombus founded in aspiration. Eventually, the obstruction resolved after repeated intracoronary injection of nitroglycerin (1 mg) (Fig. 4). The fact that intracoronary nitroglycerin led to resolution of coronary obstruction confirmed the diagnosis of CAS. Under systemic diltiazem infusion, subsequent RFCA was preformed successfully. After the procedure, the patient recovered without any neurologic deficit, and CAS has never recurred with isosorbide mononitrate treatment during follow-up.

## Discussion and conclusions

Most of the AF procedure related CAS were reported to occur during energy application and transseptal procedure in ablation [1, 2, 4–8], unlike our patient who also developed CAS during LAAC. To our knowledge, this is the first case report of CAS related to AF hybrid procedure.

The underlying pathophysiology of CAS could be the hyperreactivity of the coronary artery and increased activity of vasoconstrictive factors such as acetylcholine, inflammatory, and imbalance in autonomic tone [5]. The potential mechanism behind the CAS in this case could be the change in autonomic tone. Human histopathologic studies revealed that atrium contained ganglionated plexuses (GP) that regulate cardiac autonomic balance [9]. Left atrial (LA) manipulation and ablation injury could stimulate the GP, which would cause a sudden increase in autonomic tone and cause CAS [10, 11]. Iso et al. [12] reported that persistent AF had a higher vagal response elicited by high-frequency stimulation of the LA GP than paroxysmal AF. Additionally, most patients with a serious condition during the CAS had persistent AF [2], which indicates that the higher autonomic nerve activity in patients would cause more severe CAS. Innervation of vagal fibers in right coronary artery is greater than that in left coronary artery, and CAS occurred mostly in right coronary artery like our case [2, 4, 13]. This case further reveals the role of autonomic tone in CAS associated with AF procedure. Thus, we argue that the imbalance in autonomic tone caused by LA manipulation and ablation could be the major reason for this complication.

Although no air embolism was observed in coronary angiography, we cannot entirely exclude it, as small air bubbles would be absorbed before performing angiography. Lesh et al. [14] reported a case of air embolism during AF ablation, and air embolism resolved after administration of nitroglycerin, oxygen, and forceful injections of contrast medium in several minutes. Coronary air embolism is a well-recognized complication of AF ablation, especially under general anesthesia [15]. Air emboli can enter right coronary artery through transseptal sheath in the supine patient, as the right coronary artery ostium was in a superior position [16], which can partly explain ST-segment elevation in II, III, and aVF in our patient.

Additionally, our patient had hypertension and peripheral artery disease, which would contribute to endothelial dysfunction. The hyperreactivity of the coronary artery in response to vasoconstrictive factors owning to endothelial dysfunction would be another factor contributing to CAS [5]. Therefore, more attention should be paid to this complication in AF patients whose condition is complicated by hyperreactivity of the coronary artery such as coronary atherosclerosis, drug-eluting stent implantation, and smoking.

Several cases have reported that transmural direct thermal injury may also be responsible for CAS [2, 8, 17]. In our case, coronary angiogram revealed the obstruction in the right coronary artery, and the obstruction was resolved after the administration of intracoronary nitroglycerin. Such a diffuse reversible obstruction made it unlikely that the transmural direct thermal injury was the mechanism for the patient’s CAS.

We reported a clinical case of AF hybrid procedure related CAS. This complication could be induced by multiple factors, especially the acute imbalance of autonomic tone due to the stimulation of GP. The recognition of this rare but potentially life-threatening complication is important to improve the practice of AF hybrid procedure.

## Data Availability

The data underlying this article will be shared on reasonable request to the corresponding author.

## Abbreviations

LAAC: Left atrial appendage closure
RFCA: Radiofrequency catheter ablation
AF: Atrial fibrillation
CAS: Coronary artery spasm
BP: Blood pressure
HR: Heart rate
TEE: Transoesophageal echocardiography
GP: Ganglionated plexuses
LA: Left atrial
